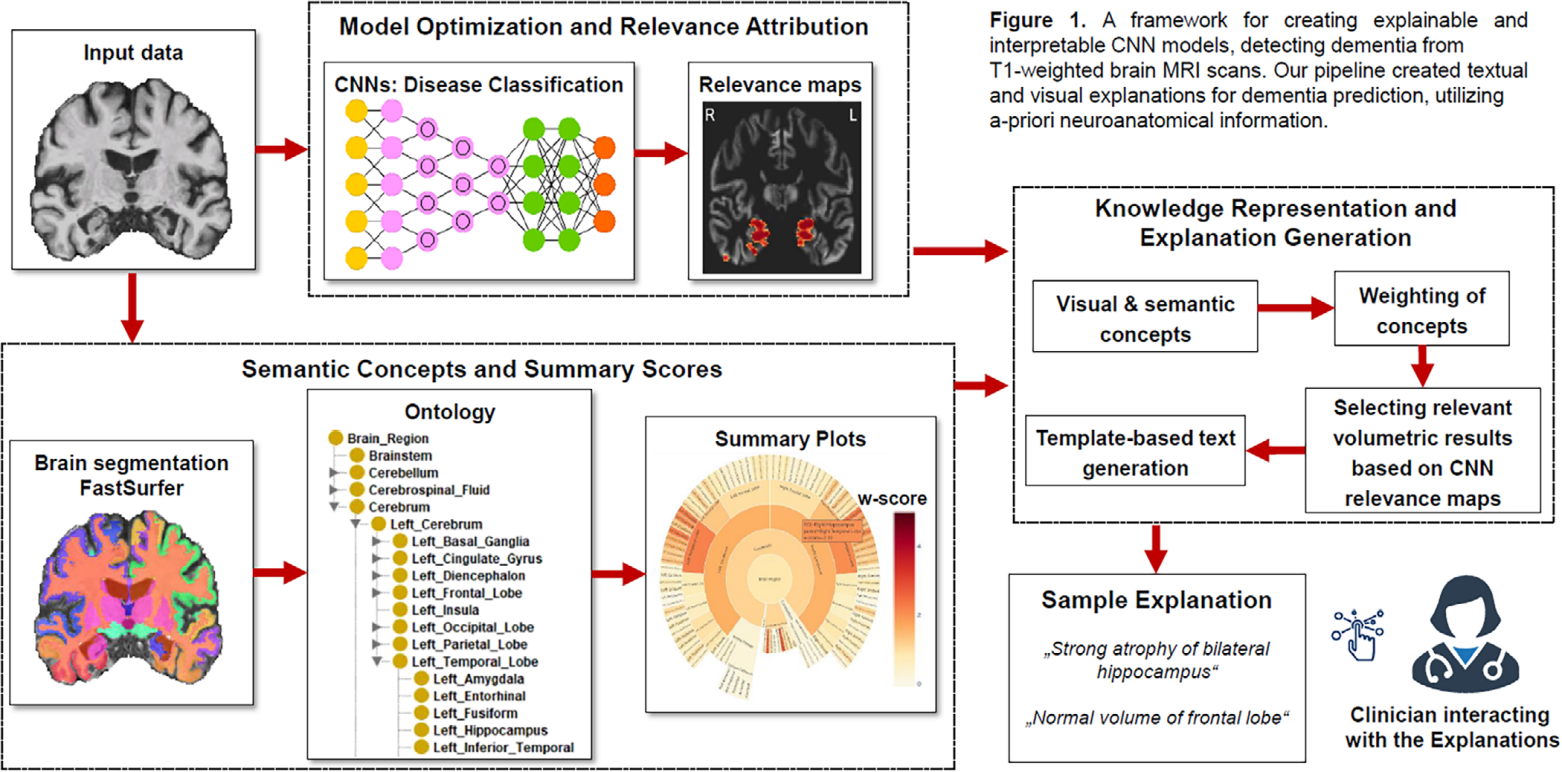# An explainable framework for convolutional neural networks detecting dementia in MRI scans

**DOI:** 10.1002/alz.093677

**Published:** 2025-01-09

**Authors:** Devesh Singh, Alice Grazia, Martin Dyrba, Stefan Teipel

**Affiliations:** ^1^ German Center for Neurodegenerative Diseases (DZNE), Rostock/Greifswald Germany; ^2^ Department of Psychosomatic Medicine, Rostock University Medical Center, Rostock Germany

## Abstract

**Background:**

With a global ageing population, there is an increasing demand for fast and reliable early diagnosis of individuals. Convolutional neural networks (CNNs) have an immense potential in assisting clinicians in diagnosing dementia. Regional atrophy patterns, which are visible in T1‐weighted MRI scans, have been consistently identified by the CNNs with high accuracy. However, there is still hesitation in the clinical adoption of CNNs, due to their black box nature. To alleviate these issues, we propose a framework for explainable CNN models to detect neurodegenerative diseases.

**Method:**

Using data from multiple data cohorts (N>3000) we trained a robust DenseNet CNN model and utilised the feature attribution method ‘layer‐wise relevance propagation’ (LRP) to highlight image features found useful by the model. The FastSurfer tool was utilised to segment individual brain scans into various anatomical region of interests (ROIs). We created an ontology model for encoding the hierarchy of and relationships between neuroanatomical structures. Using linear regression models, controlling for common covariates such as age and sex, we quantified the volumetric deviance from normal levels (w‐scores) for all ROIs. These findings were visualised as summary plots. We integrate two different sources of information about pathology in the MRI scans, 1) the CNN predictive models and 2) the volumetric atrophy analysis. An algorithmic framework was developed to combine both of them and to select the information to be included in textual explanations for the CNN’s predictions (Figure 1).

**Result:**

Our framework results in a modular pipeline where each component – attribution method, segmentation tool, ontology model, could be altered due to advancements or changes in the requirements. Our framework implements a plug‐and‐play functionality for interpreting trained CNN models. It assists an easy understanding of CNN’s prediction by generating textual and visual explanations for its findings, with variable degree of detail, focusing on specific ROIs or lobes.

**Conclusion:**

Our study provides a proof‐of‐concept of utilizing a‐priori neuroanatomical information for explaining CNNs. This prototypical implementation for generating explainable AI is going to be tested by clinicians to evaluate its utility for assisting the diagnosis of dementia. We also plan to extend our framework to other neurodegenerative diseases.